# DJ-1 modulates the unfolded protein response and cell death via upregulation of ATF4 following ER stress

**DOI:** 10.1038/s41419-019-1354-2

**Published:** 2019-02-12

**Authors:** Jungwoo Yang, Kwang Soo Kim, Grace O. Iyirhiaro, Paul C. Marcogliese, Steve M. Callaghan, Dianbo Qu, Woo Jae Kim, Ruth S. Slack, David S. Park

**Affiliations:** 1 Department of Cellular and Molecular Medicine, University of Ottawa Brain and Mind Research Institute, University of Ottawa, Ottawa, ON K1H 8M5 Canada; 2 Department of Clinical Neurosciences, and Cell Biology and Anatomy, Hotchkiss Brain Institute, University of Calgary, Calgary, AB T2N 4N1 Canada

## Abstract

The unfolded protein response (UPR) triggered by endoplasmic reticulum (ER) stress is a feature of many neurodegenerative diseases including Alzheimer’s disease, Huntington’s disease and Parkinson’s disease (PD). Although the vast majority of PD is sporadic, mutations in a number of genes including *PARK7* which encodes the protein DJ-1 have been linked to early-onset, familial PD. In this regard, both PD of sporadic and genetic origins exhibit markers of ER stress-induced UPR. However, the relationship between pathogenic mutations in *PARK7* and ER stress-induced UPR in PD pathogenesis remains unclear. In most contexts, DJ-1 has been shown to protect against neuronal injury. However, we find that DJ-1 deficiency ameliorates death in the context of acute ER stress in vitro and in vivo. DJ-1 loss decreases protein and transcript levels of ATF4, a transcription factor critical to the ER response and reduces the levels of CHOP and BiP, its downstream effectors. The converse is observed with DJ-1 over-expression. Importantly, we find that over-expression of wild-type and PD-associated mutant form of *PARK7*^*L166P*^, enhances ER stress-induced neuronal death by regulating ATF4 transcription and translation. Our results demonstrate a previously unreported role for wild-type and mutant DJ-1 in the regulation of UPR and provides a potential link to PD pathogenesis.

## Introduction

Parkinson’s disease (PD) is a progressive movement disorder characterized by loss of dopaminergic (DA) neurons in the Substantia Nigra pars compacta (SNpc). Clinical manifestations of PD include rigidity, resting tremor, bradykinesia, postural instability, and cognitive dysfunction^1,2^. The majority of PD cases are sporadic, however, ~10% of PD is familial^3,4^. DJ-1 encoded by *PARK7* may be important in both sporadic^5–7^ and familial PD^8^. For example, in sporadic PD, DJ-1 shows increased oxidation^9^, and is elevated in patient brain and spinal fluid^6,7^. Similarly, mutations in *PARK7* account for ~1% of autosomal-recessive familial PD cases. Recessive mutations such as p.M26I, p.E64D and p.L166P in *PARK7* are pathogenic^8,10^. A subset of *DJ-1* null mice on a heavily backcrossed C57BL/6N background exhibit neurodegeneration^11^. While these studies implicate DJ-1 in sporadic and familial PD, the underlying mechanism connecting it to both forms of PD is unclear. One potential mechanism connecting DJ-1 to both forms of PD is the activation of the unfolded protein response (UPR) pathway induced by endoplasmic reticulum (ER) stress. Previous studies have shown that other PD related genes are associated with the UPR pathway. For example, *LRRK2*-linked PD mutations are associated with ER in DA neurons of PD patients^12^. LRRK2 also upregulates GRP78, a key survival molecule during ER stress^13^. In *Drosophila* models of PD, mutations in recessive PD genes: Parkin and Pink1 induce ER stress through activating PERK^14^. ER stress-induced activation of the UPR has been demonstrated in the brains of sporadic PD patients and in animal models of familial PD^15^.

ER stress-induced UPR is characterized by increased phosphorylation of protein kinase R (PKR)-like endoplasmic reticulum kinase (P-PERK), its downstream substrate, eukaryotic initiation factor 2α (P-eIF2α) and activating transcription factor 4 (ATF4)^16^. ATF4, a member of the ATF/CREB family of basic leucine zipper transcriptional factor, is upregulated by elevated P-eIF2α in cellular stress conditions, such as viral infection, oxidative stress, and ER stress^17^.

Pro-survival and pro-apoptotic roles have been reported for ATF4 in models of ER stress-induced cell death and PD^16,18,19^. In the context of PD, increase in ATF4 is observed in neuromelanin positive neurons in the SNpc in a subset of PD patients and in cellular models of PD^18^. Over-expression of ATF4 was found to promote cell survival while its downregulation enhanced death^18^. On the contrary, over-expression of ATF4 has been shown to induce DA neurons loss in a rat model of PD indicating a pro-apoptotic role for ATF4 in PD^20^. While seemingly conflicting, together these studies suggest that the activation of ER stress-induced UPR signaling can trigger adaptive responses that may be protective or detrimental to vulnerable neurons in PD. However, it is unclear how PD-linked genes such as *PARK7* and their pathogenic mutations modulate ER stress-induced responses. Here, we explore the role of DJ-1 in the UPR response following ER stress. We show that DJ-1 regulates ATF4 signaling with an unexpected and previously undefined role in neuronal survival following acute ER stress.

## Results

### DJ-1 deficiency downregulates basal ATF4 levels

**Fig. 1:**
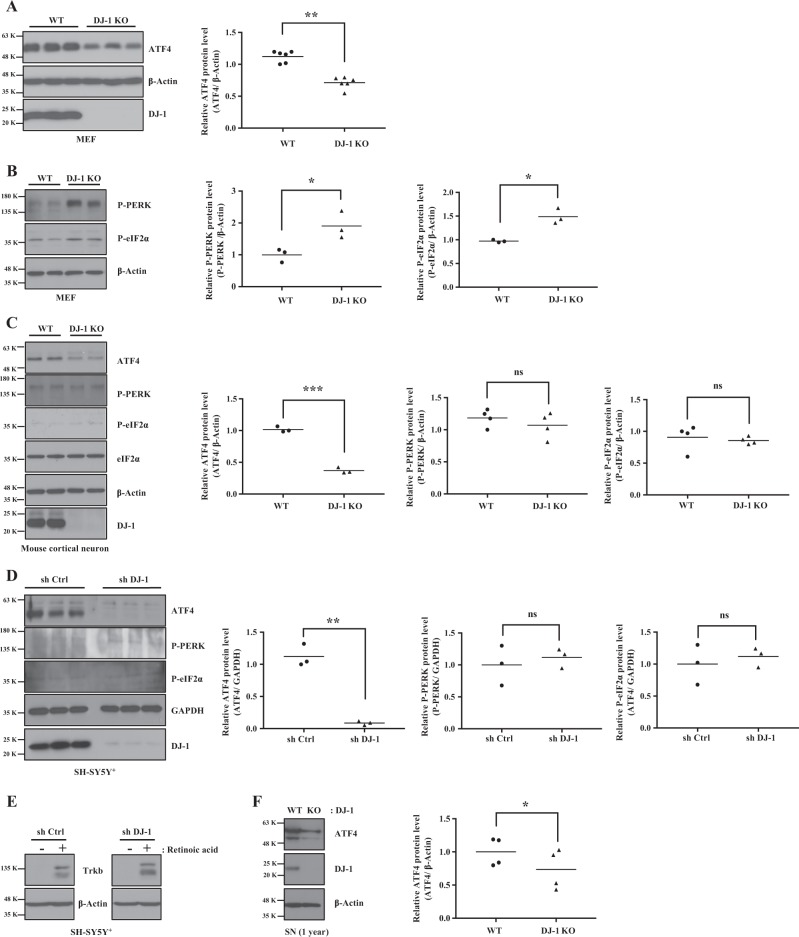
DJ-1 deficiency downregulates the ATF4 protein expression. **a**–**c** Left panels: WT and DJ-1 KO cells. Blots were probed with the indicated antibodies and quantified by Image J. Right panels of **a**–**c**: Data were obtained from the same conditions and averaged to compare ATF4, P-PERK, or P-eIF2α protein levels between DJ-1 WT and KO in **a** MEFs (**p < 0.01, paired two-tailed *t*-test, *n* = 6), **b** MEFs (*p < 0.05, paired two-tailed *t*-test, *n* = 3 for P-PERK and *p < 0.05, paired two-tailed *t*-test, *n* = 3 for P-eIF2α), **c** mouse cortical neurons (***p < 0.001, paired two-tailed *t*-test, *n* = 3 for ATF4 and ns: *p* > 0.05, paired two-tailed *t*-test, *n* = 4 for P-PERK or P-eIF2α) after normalization with β-Actin. β-Actin was used as a loading control. **d** Left panel: After treatment of retinoic acid for 7 days, SH-SY5Y^+^ with stable expression of control shRNA vector (sh Ctrl) or DJ-1 shRNA vector (sh DJ-1) were lysed and immunoblotted by indicated antibodies and quantified by Image J. Right panels of **d**: Data were obtained from the same conditions and averaged to compare ATF4 (**p < 0.01, paired two-tailed *t*-test, *n* = 3), P-PERK or P-eIF2α (*p* > 0.05, paired two-tailed *t*-test, *n* = 3) protein levels between sh Ctrl and sh DJ-1 in SH-SY5Y^+^ cells after normalization by GAPDH. GAPDH were used as a loading control. **e** After treatment of retinoic acid (10 µM) or dimethyl sulfoxide (DMSO) as a vehicle control for 7 days, SH-SY5Y^+^ cells with stable expression of control shRNA vector (sh Ctrl) and DJ-1 shRNA vector (sh DJ-1) were lysed and immunoblotted by TrkB. β-Actin were used as a loading control. **f** Left panel: WT and DJ-1 KO tissues were collected from SN of 1-year-old mice. Blots were probed with the indicated antibodies and quantified by Image J. Right panels of **f**: Data were obtained from the same conditions and averaged to compare ATF4 (*p < 0.05, paired two-tailed *t*-test, *n* = 4) protein levels between WT and DJ-1 KO in SN mice brain. β-Actin were used as a loading control

ER stress-induced UPR signaling in post-mortem brains of patients and animal models of PD has been documented^16^. However, whether or how PD genes modulate UPR remains unknown. Hence, we first tested whether there were perturbations in ATF4, a key regulator of UPR, in DJ-1 wild-type (WT) and knock-out (KO) mouse embryonic fibroblasts (MEFs). Under basal conditions, ATF4 protein level was significantly reduced in DJ-1 KO MEFs vs controls (Fig. 1a). Following ER stress, PERK and eIF2α are increasingly phosphorylated resulting in increased ATF4 expression^21^. The reduction in ATF4 protein thus prompted us to examine whether there were corresponding changes in its upstream regulators. Surprisingly, phosphorylated PERK and eIF2α were significantly increased in DJ-1 KO MEFs vs WT controls (Fig. 1b). To determine whether this phenomenon was cell-specific, we conducted similar experiments in primary mouse cortical neurons, from DJ-1 WT and KO mice. We also examined differentiated human neuroblastoma cells, SH-SY5Y(SH-SY5Y^+^) cells with shRNA-mediated DJ-1 knock-down (KD). Consistent with our results in MEFs, ATF4 protein levels were significantly reduced in DJ-1 KO neurons (Fig. 1c). Similarly, ATF4 protein was dramatically reduced following KD of DJ-1 in the SH-SY5Y^+^ cells (Fig. 1d). The differentiation status of SH-SY5Y^+^ cells was verified by TrkB expression (Fig. 1e). Unlike in MEFs, the levels of P-PERK and P-eIF2α remained unchanged in DJ-1 KO neurons (Fig. 1c) and DJ-1 KD SH-SY5Y^+^ cells (Fig. 1d). This result suggested that the downregulation of ATF4 in the absence of DJ-1 maybe independent of its upstream regulators. To confirm the physiological relevance of these findings, we examined ATF4 protein level in the substantia nigra (SN) in aged (one year old) DJ-1 KO and WT mice. Consistent with our in vitro data, ATF4 levels were significantly reduced in the SN of DJ-1 KO mice vs WT controls (Fig. 1f).

**Fig. 2:**
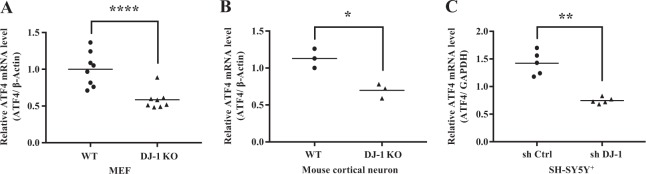
DJ-1 deficiency downregulates the transcription level of ATF4. **a**–**c** Total RNAs was prepared from **a** MEFs (****p *<* 0.0001, Paired two-tailed *t*-test, *n* = 8), **b** mouse cortical neuron (*p *<* 0.05, Paired two-tailed *t*-test, *n* = 3) and **c** SH-SY5Y^+^ cells (**p *<* 0.01, paired two-tailed *t*-test, *n* = 5). cDNA was prepared using oligo dT primers. Synthesized cDNAs were used as templates. mRNA level of ATF4, β-Actin, or GAPDH were determined by real-time PCR

We next asked whether the decrease in ATF4 protein in DJ-1 KO or KD cells was associated with changes in its transcript levels. ATF4 transcript levels, similar to the above protein analysis, were significantly reduced in DJ-1 KO MEFs, cortical neurons, and DJ-1 KD SH-SY5Y^+^ cells (Fig. 2). Together, our data show that in MEFs and neuronal cells, DJ-1 deficiency leads to a downregulation of ATF4 protein/transcript.

### DJ-1 deficiency inhibits ER stress-induced ATF4 protein up-regulation

**Fig. 3:**
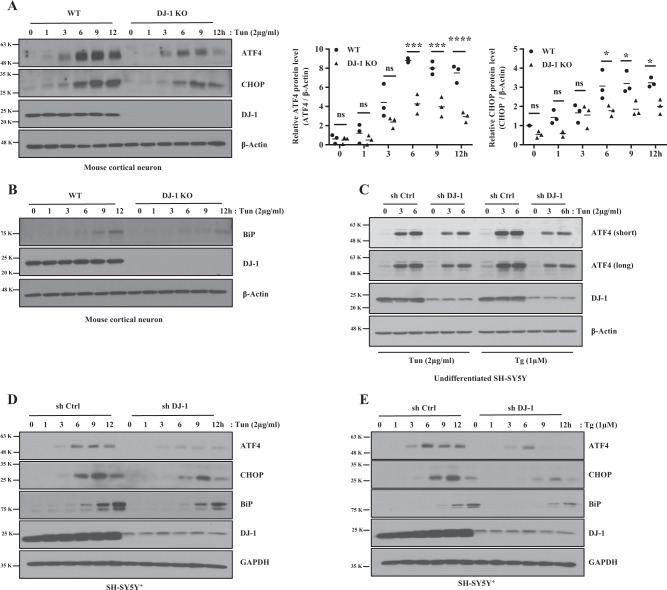
DJ-1 deficiency downregulates ER stress-induced ATF4 protein expression and its downstream factors. **a** Left panel: Mouse cortical neurons DJ-1 WT and KO were collected after treatment with DMSO (0 h) as vehicle control or Tun for up to 12 h. Blots were probed with the indicated antibodies and quantified by Image J and graphed by Prism 7. Right panels of **a**: Data of **a** were averaged to compare ATF4 (***p < 0.001, ****p < 0.0001, two-way ANOVA with Tukey post-test, *n* = 3) or CHOP (*p < 0.05, two-way ANOVA with Tukey post-test, *n* = 3) protein levels between DJ-1 WT and KO for up to 12 h after normalization with β-Actin. **b** Experiments are conducted as in **a**. Blots were probed with the indicated antibodies. **c** Undifferentiated SH-SY5Y cells stably expressing sh Ctrl and sh DJ-1 were collected after treatment with DMSO (0 h) as vehicle control, Tun or Tg for 3 h or 6 h. Blots were probed with the indicated antibodies. **d**, **e** SH-SY5Y^+^ cells stably expressing sh Ctrl and sh DJ-1 were collected after treatment with DMSO (0 h), Tun or Tg for up to 12 h. Blots were probed with the indicated antibodies

ER stress is a known inducer of ATF4 protein expression^22^. Accordingly, we tested the effect of DJ-1 on ATF4 induction in response to chemically mediated ER stress. Tunicamycin (Tun) and Thapsigargin (Tg) are known inducers of ER stress^21^. As expected, ATF4 protein was induced in WT cortical neurons and MEFs following Tun or Tg treatment. However, in the absence of DJ-1, the expected increase in ATF4 was reduced for up to 12 h (h) vs WT controls (Fig. 3a, Supplementary data 1A,B). Similar results were observed with DJ-1 KD in undifferentiated SH-SY5Y (Fig. 3c) and SH-SY5Y^+^ cells (Fig. 3d, e). Increase in ATF4 upregulates C/EBP homologous protein (CHOP) and immunoglobulin heavy-chain binding protein (BiP, an ER chaperone)^23,24^. Compared with control, induction of CHOP and BiP were significantly attenuated in DJ-1 KO neurons, MEFs and KD SH-SY5Y^+^ cells (Fig. 3a, b, d, e and Supplementary data 1). Interestingly, we found that P-PERK and P-eIF2α were more activated in DJ-1 KO MEFs (Supplementary data 1A). This increase indicates that other parallel ER stress pathways may be activated. Therefore, we examined other UPR pathways including inositol-requiring enzyme-1 (IRE1)/XBP-1 and ATF6. Activation of IRE1 or ATF6 by ER stress induces IRE1 auto-phosphorylation and endonuclease activity resulting in spliceosome-independent splicing of XBP-1 mRNA and cleaved ATF6 translocation to the nucleus and function as a transcriptional factor, respectively^25^.

We found that DJ-1 KO MEFs displayed increased P-IRE1, that correlated with a rapid splicing of XBP-1 mRNA during ER stress and increased cleaved ATF6 (Supplementary data 2A, B, D), indicating that DJ-1 deficiency itself triggers ER stress in MEFs. Intriguingly, we did not observe this in cortical neurons (Supplementary data 2C).

**Fig. 4:**
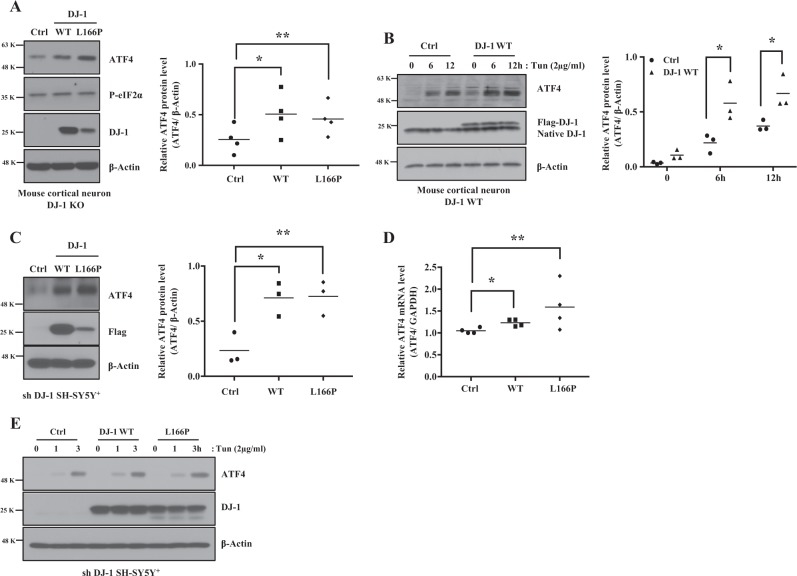
Effects of over-expressed DJ-1 WT and pathogenic mutant L166P on basal or ER stress-induced ATF4 expression. **a** DJ-1 KO mouse cortical neurons were infected with adenovirus-expressing GFP (as a reporter) plus Flag tagged DJ-1 WT, or GFP plus Flag tagged DJ-1 L166P, or GFP plus pcDNA3 (empty vector) as a control for 30 h, then cells were collected. Blots were probed with the indicated antibodies and quantified by Image J and graphed by Prism 7 (*p < 0.05, **p < 0.01, one-way ANOVA with Tukey post-test, *n* = 4). **b** DJ-1 WT mouse cortical neurons were infected with the same adenovirus constructs as in **a** and treated with Tun (2 μg/ml) for 6 or 12 h and DMSO (0 h). Blots were probed with the indicated antibodies and quantified by Image J and graphed by Prism 7 (*p < 0.05, two-way ANOVA with Tukey post-test, *n* = 3). **c** Experiments were conducted as in A in DJ-1 KD SH-SY5Y^+^ cells. Blots were probed with the indicated antibodies and quantified by Image J and graphed by Prism 7 (C, *p < 0.05, **p < 0.01, one-way ANOVA with Tukey post-test, *n* = 3). **d** Experiments are conducted as in **a** in DJ-1 KD SH-SY5Y^+^ cells. Total RNAs were prepared using oligo dT primers. Synthesized cDNAs were used as templates. mRNA levels of ATF4 or GAPDH were determined by real-time PCR (*p < 0.05, **p < 0.01, one-way ANOVA with Tukey post-test, *n* = 4). **e** Experiments are conducted as in A in DJ-1 KD SH-SY5Y^+^ cells, then treated with Tun (2 μg/ml) for 1 or 3 h and DMSO (0 h) as vehicle control. Blots were probed with the indicated antibodies

To determine how PD linked DJ-1 mutations affect ATF4 protein expression under basal and ER stress conditions, we infected cortical neurons and SH-SY5Y^+^ cells with recombinant-adenovirus-expressing Flag-DJ-1 WT, Flag-DJ-1 L166P, or pcDNA3 (empty vector) as a control for 30 h following infection. Exogenous Flag-DJ-1 WT or Flag-DJ-1 L166P are expressed at levels of 106% and 50.4%, respectively, compared to native DJ-1 in cortical neurons. Both DJ-1 and L166P samples expressed identical levels of signal (Supplementary data 3C, D). ATF4 protein was significantly increased in DJ-1 KO cortical neurons infected with WT or L166P (Fig. 4a) under basal conditions. With ER stress, expression of DJ-1 WT also significantly increased ATF4 protein in WT cortical neurons (Fig. 4b). Similar results were observed in SH-SY5Y^+^ cells upon DJ-1 KD and MEFs (Fig. 4c and Supplementary data 3A, B). ATF4 mRNA and protein levels were increased in SH-SY5Y^+^ cells infected with WT and L166P DJ-1 basally or under ER stress conditions (Fig. 4d, e). Together, our data show that DJ-1 expression or loss increases or decreases ATF4 protein/transcript levels, respectively.

### DJ-1 directly interacts with ATF4 mRNA

To further explore the mechanism by which DJ-1 regulates ATF4, we queried whether DJ-1 and ATF4 physically interact. We performed co-immunoprecipitation, but found no evidence of physical interaction between the two proteins (Supplementary data 4A–D).

**Fig. 5:**
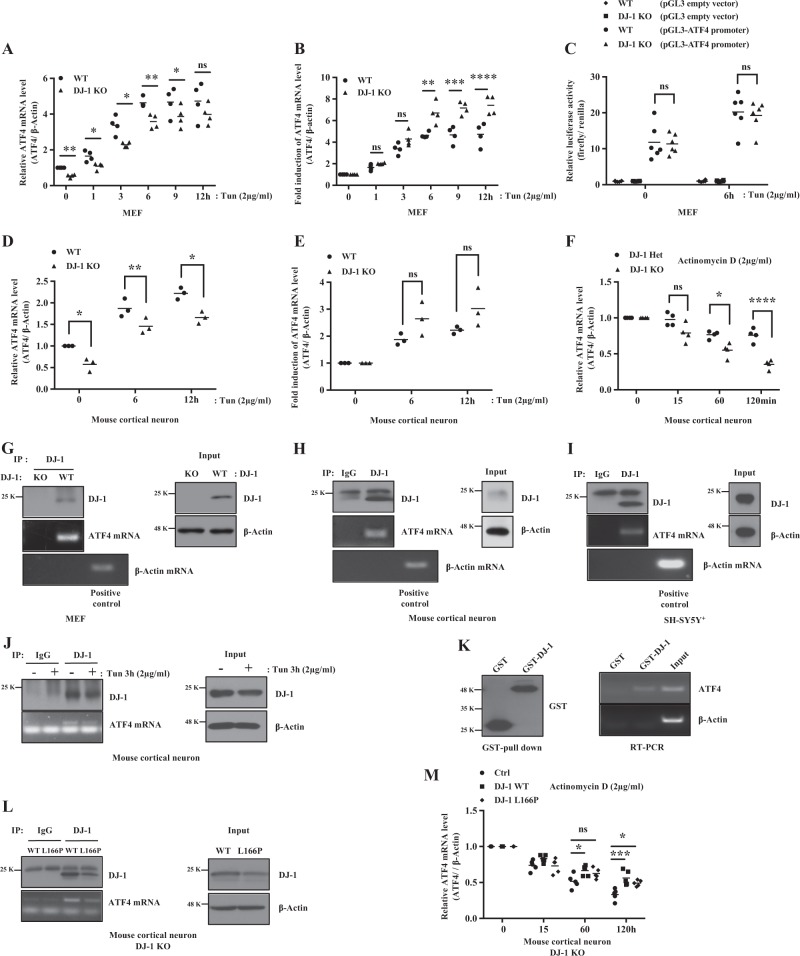
DJ-1 regulates mRNA stability of ATF4 through binding with ATF4 mRNA but not transcription level of ATF4. cDNA was prepared using oligo dT primers. Synthesized cDNAs were used as templates. mRNA levels of ATF4 and β-actin were determined by real-time PCR in MEFs (**a**, **b**, *p < 0.05, **p < 0.01, ***p < 0.001, ****p < 0.0001, two-way ANOVA with Tukey post-test, *n* = 4) and mouse cortical neurons (**d**, **e**, *p < 0.05, **p < 0.01, two-way ANOVA with Tukey post-test, *n* = 3). Data are averages from three or four independent experiments in MEFs or mouse cortical neurons. **c** Luciferase assay was performed by using DJ-1 WT and KO MEFs after transfection with pGL3 (empty vector) and pGL3 fused with ATF4 promoter vector after treatment with Tun for 6 h. Data are averages from six independent experiments. **f** Heterozygous DJ-1 and DJ-1 KO mouse cortical neuron were collected after treatment with DMSO (0 h) as a vehicle control or actinomycin D for 15, 60, or 120 min. Data are averages from four independent experiments (*p < 0.05, ****p < 0.0001, two-way ANOVA with Tukey post-test, *n* = 4). RIP assay was performed by using MEFs (**g**), mouse cortical neuron (**h**, **j**) treated with or without Tun for 3 h or DMSO (0 h) as vehicle control, and SH-SY5Y^+^ (**i**) cells. DJ-1 protein was isolated by immunoprecipitation (IP) using anti-goat-DJ-1 antibody and protein G beads. DJ-1-associated RNA was isolated with Trizol and amplified using oligo dT primers for reverse transcription and ATF4 or β-Actin primers for PCR. An input was detected by immunoblotting with the DJ-1 or β-Actin antibody. **k** Recombinant GST or GST-DJ-1 immobilized on glutathione-Separose beads pull-down ATF4 mRNA. Pulldowns were immunoblotted with anti-GST antibody or detected by RT-PCR for ATF4 mRNA. **l** RIP assay is conducted as in **h** using DJ-1 KO mouse cortical neurons infected with adenovirus-expressing DJ-1 WT, L166P, or pcDNA3 (empty vector) as control for 30 h. An input was detected by immunoblotting with the DJ-1 or β-Actin antibody. **m** Analysis of ATF4 mRNA stability (*p < 0.05, ***p < 0.001, two-way ANOVA with Tukey post-test, *n* = 5) was conducted as in **f** using DJ-1 KO mouse cortical neurons infected with adenovirus-expressing DJ-1 WT, L166P, or pcDNA3 (empty vector) as control

DJ-1 is a multi-functional protein known to act as a transcription factor, an antioxidant response activator, a weak scavenger of reactive oxygen species (ROS), a chaperone, a protease, and an RNA binding protein^26,27^. Therefore, we examined whether DJ-1 is involved in the transcriptional regulation of ATF4. We analyzed ATF4 transcription in MEFs and cortical neurons in response to Tun by real-time PCR. In response to ER stress, the relative mRNA levels of ATF4 was greater in DJ-1 WT MEFs (Fig. 5a) and cortical neurons (Fig. 5d), vs DJ-1 KO cells. However, the induction (fold change) in ATF4 mRNA in DJ-1 KO MEFs and cortical neurons was increased, compared to DJ-1 WT cells (Fig. 5b, e). These results suggest that DJ-1 does not regulate ATF4 at a transcriptional level. To confirm this, we performed luciferase assays with a reporter plasmid harboring *Firefly* luciferase gene and *Renilla* luciferase gene as an internal control. There is no difference in ATF4 promoter activity in DJ-1 WT or KO cells in the presence or absence of ER stress (Fig. 5c). These results suggest that DJ-1 does not regulate the transcription of ATF4 under ER stress.

We next examined whether DJ-1 influences ATF4 mRNA stability. To this end, cortical neurons were treated with actinomycin D to inhibit gene transcription^28^. We found that ATF4 mRNA stability was greater in DJ-1 Het neurons vs DJ-1 KO cells (Fig. 5f). Previous studies suggest that DJ-1 can bind ATF4 mRNA^26^. We tested this by performing RNA immunoprecipitation assay in MEFs, cortical neurons and SH-SY5Y^+^ cells. Endogenous DJ-1 was immunoprecipitated and analyzed for association with ATF4 mRNA. DJ-1 bound to ATF4 mRNA in MEFs (Fig. 5g), cortical neurons (Fig. 5h) and SH-SY5Y^+^ cells (Fig. 5i), but not to β-actin mRNA under basal conditions. DJ-1 protein also physically interacted with ATF4 mRNA under ER stress in cortical neurons (Fig. 5j). The interaction did not change upon different conditions. To further ascertain whether DJ-1 directly interacts with ATF4 mRNA, a glutathione *S*-transferase (GST) pull-down assay was performed with bacterially generated GST-DJ-1 or GST as control. Total RNA was isolated from mouse whole brains. ATF4 mRNA was detected by RT-PCR in GST-DJ-1 but not in GST alone, supporting the model that DJ-1 directly interacts with ATF4 mRNA (Fig. 5k).

We found that mutant L166P binds ATF4 mRNA similar to WT DJ-1 (Fig. 5l). ATF4 mRNA stability was increased with over-expression of WT and L166P DJ-1, vs empty vector in DJ-1 KO neurons (Fig. 5M). Together, these results suggest that DJ-1 regulates ATF4 levels by stabilizing its mRNA through a direct protein–mRNA interaction.

### DJ-1 in ER stress-induced cell death

**Fig. 6:**
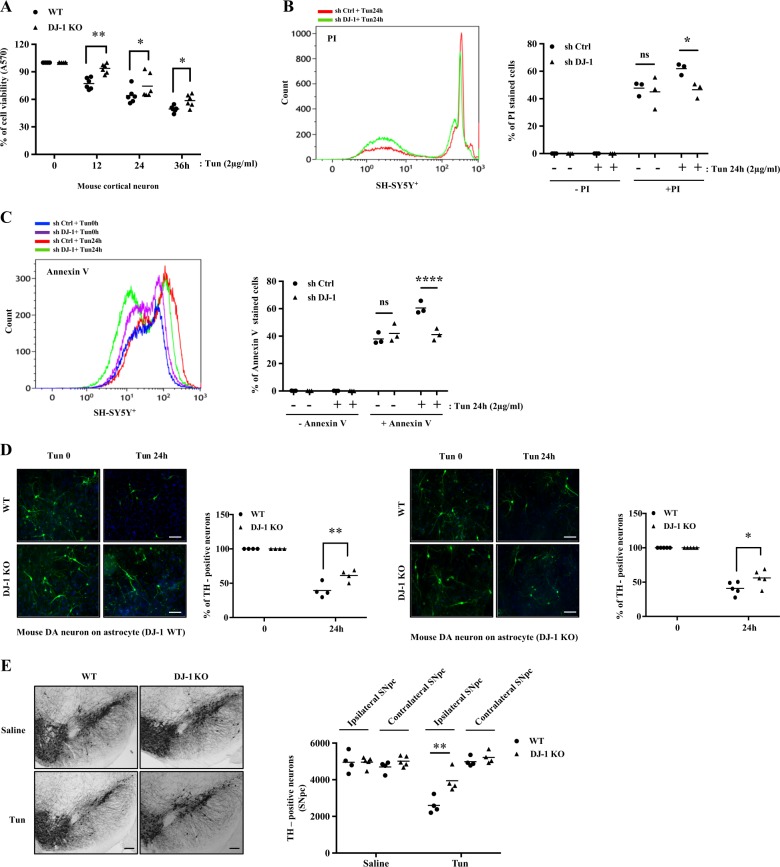
DJ-1 deficiency inhibits ER stress-induced cell death. **a** DJ-1 WT and KO mouse cortical neurons were treated with Tun for 12, 24, or 36 h at the concentration indicated. Cell viability was detected by MTT assay. Cell viability was expressed as absorbance 570 nm relative to that of 0 h (DMSO-treated controls) (*p < 0.05, **p < 0.01, two-way ANOVA with Tukey post-test, *n* = 6). SH-SY5Y^+^ cells were stably expressed either control shRNA vector or DJ-1 shRNA vector treated with or without Tun (24 h) at indicated concentration and analyzed for induction of cell death and apoptosis by measuring PI (**b**) or Annexin-V (**c**) staining followed by flow cytometry. Induction of PI (*p < 0.05, two-way ANOVA with Tukey post-test, *n* = 3) or Annexin-V (****p < 0.0001, two-way ANOVA with Tukey post-test, *n* = 3) staining in three independent experiments by Tun 24 h was quantified with Kaluza and graphed using Prism 7 in SH-SY5Y^+^ cells (**b**, **c**). **d** Upon the cortical astrocyte primary culture from postnatal day 0 to 5 days in DJ-1 WT or KO mice, the mesencephalic dopamine neuron culture was performed from brains from 0 to 2 days animals from DJ-1 WT or KO mice. The mouse postnatal mesencephalic dopamine neurons on a cortical astrocyte layer were treated with Tun (24 h) at indicated concentration, then cells were fixed with 4% PFA and assessed by direct microscopy and TH^+^ stained cells counted (*p < 0.05, **p < 0.01, two-way ANOVA with Tukey post-test), *n* = 4 (DJ-1 WT astrocytes) and *n* *=* 5 (DJ-1 KO astrocytes) (Scale bars, 200 μm). **e** Tun or 0.9% saline (control) was stereotaxically administrated above the SN of 1 year old DJ-1 WT and KO mice. Sections of the SN at the level of the medial terminal nucleus were prepared 7 days post injection. Sections were immunostained to detect TH-positive neurons. Representative pictures are DAB-stained TH-positive neurons in SN and performed quantification using Stereo Investigator software (**p < 0.01, two-way ANOVA with Tukey post-test, *n* = 4, 5 animals per group) (Scale bars, 100 μm)

We next investigated whether DJ-1 deficiency mediates cell death induced by ER stress. Accordingly, we examined cell survival using 3-(4,5-dimethylthiazol-2-yl)-2,5-diphenyltetrazolium bromide (MTT) assay^29^ in DJ-1 WT and KO cortical neurons after Tun treatment. In the absence of DJ-1, cell viability was significantly increased after treatment with Tun, vs WT (Fig. 6a). We also assessed cell death and apoptosis using propidium iodide (PI) and annexin-V staining, respectively, in control shRNA and DJ-1 KD SH-SY5Y^+^ cells treated with Tun. Our analysis showed significantly less PI-positive staining upon DJ-1 KD following Tun treatment, vs control shRNA (Fig. 6b). Annexin-V positive staining was reduced in the DJ-1 KD SH-SY5Y^+^ cells after Tun treatment, vs control shRNA (Fig. 6c). Similar results were obtained in MEFs (Supplementary data 5A).

These results were unexpected given that DJ-1 is considered a pro-survival gene. We queried whether our results might be different in DA neurons. Accordingly, we examined the effect of DJ-1 deficiency in postnatal mesencephalic dopamine neuron cultures grown on an astrocyte monolayer. WT or DJ-1 KO postnatal mesencephalic dopamine neurons were grown on a monolayer of either DJ-1 WT or KO cortical astrocytes and treated with Tun. Consistent with our results in cortical neurons and SH-SY5Y^+^ cells, we found that DJ-1 KO cells were less sensitive to Tun-induced cell death vs WT (Fig. 6d). We also examined the effects of DJ-1 deficiency on DA neuron loss in vivo in mice injected Tun using TH^+^ or cresyl violet staining. DJ-1 deficiency significantly protected DA neuronal loss, compared to WT control mice as measured by stereology (Fig. 6e, Supplementary data 5C).

**Fig. 7:**
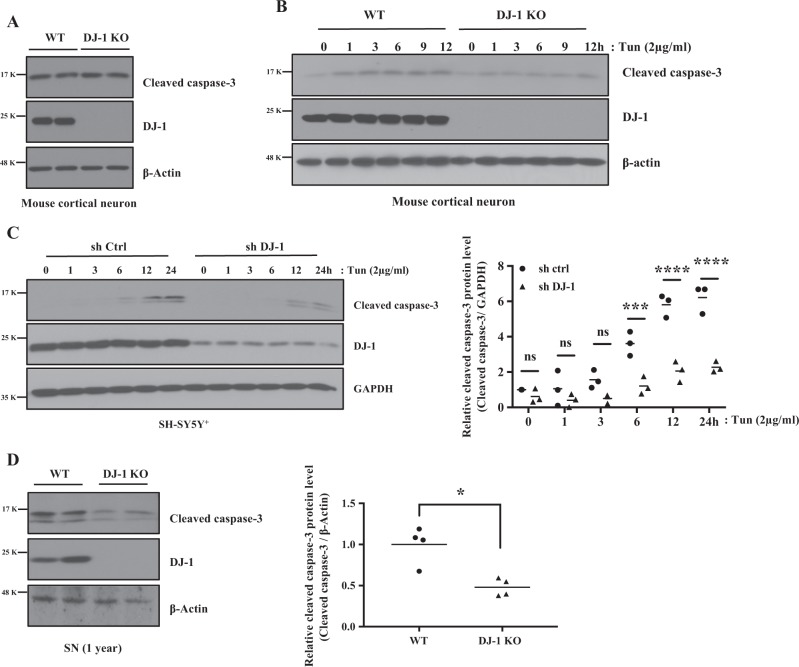
DJ-1 modulates ER stress-induced cleaved caspase-3 protein expression level. **a** DJ-1 WT and KO mouse cortical neurons were collected, and  three independent experiments were conducted. Blots were probed with the indicated antibodies. **b**, **c** Three independent experiments were conducted as in A after treatment with DMSO (0 h) as vehicle control or Tun for up to 12 h (**b**) or 24 h (**c**). Blots were probed with the indicated antibodies and graphed by Prism 7 (***p < 0.001, ****p < 0.0001, two-way ANOVA with Tukey post-test, *n* = 3). **d** DJ-1 WT and KO brain tissues were collected from SN of 1-year-old mice. Blots were probed with the indicated antibodies and quantified by Image J and graphed by Prism 7. Right panel of **d**: Data were obtained from the same conditions and averaged to compare cleaved caspase-3 (*p *<* 0.05, paired two-tailed *t*-test, *n* = 4) protein levels between DJ-1 WT and KO in SN mice brain. β-Actin were used as a loading control

We next interrogated the effect of DJ-1 deficiency on proximal death signals such as caspase-3, a critical executioner of apoptosis^30^, in response to ER stress. While basal levels remained identical (Fig. 7a), DJ-1 KO cortical neurons and DJ-1 KD in SH-SY5Y^+^ cells exhibited significantly attenuated cleaved caspase-3 protein level vs control upon ER stress (Fig. 7b, c). Interestingly, we detected lower levels of cleaved caspase-3 protein in the SN of 1 year old DJ-1 KO mouse brain, vs WT (Fig. 7d). ER stress-induced cleaved caspase-3 was similarly reduced in DJ-1 KO MEFs, vs WT, but not basally (Supplementary data 5B and 6). Together these results suggest an attenuation of proximal death effector signaling in the absence of DJ-1.

**Fig. 8:**
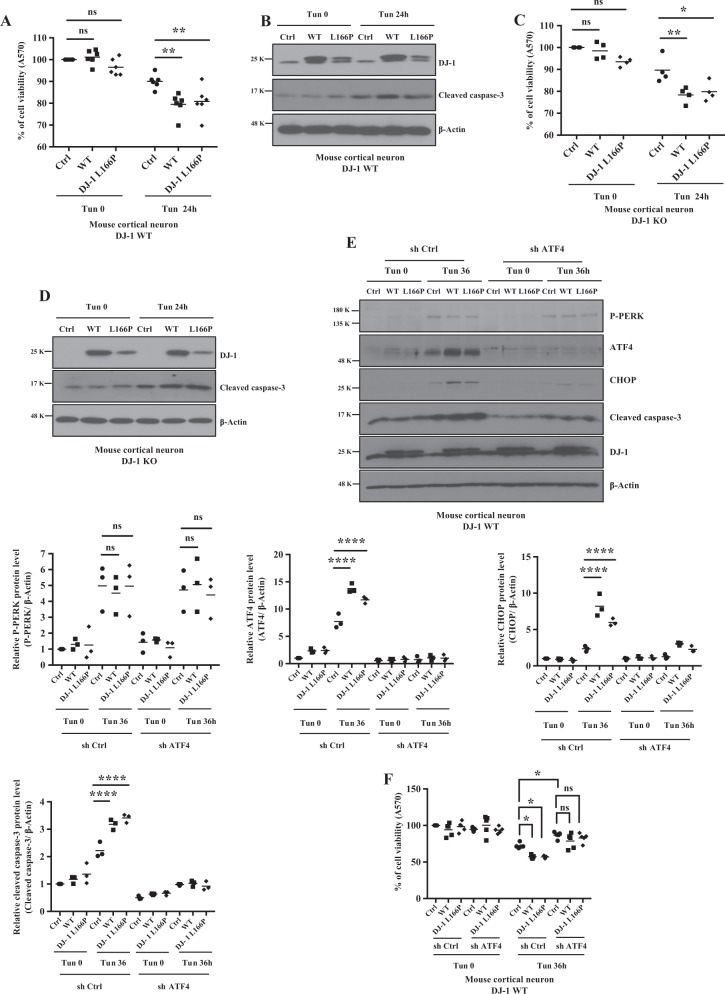
Effects of DJ-1 or pathogenic mutant L166P, and knock-down of ATF4 on ER stress-induced neuronal cell death. **a**–**d** DJ-1 WT or KO mouse cortical neuron was infected with adenovirus-expressing GFP plus Flag tagged DJ-1 WT, GFP plus Flag tagged DJ-1 L166P or GFP plus empty vector as a control and treated with Tun (2 μg/ml) for 24 h or DMSO as vehicle control. Cell viability was detected by MTT assay in DJ-1 WT (**a**, *n* = 6, **p < 0.01, two-way ANOVA with Tukey post-test) and DJ-1 KO mouse cortical neurons (**c**, *p < 0.05, **p < 0.01, two-way ANOVA with Tukey post-test, *n* = 4). Blots were probed with the indicated antibodies (**b**, **d**). **e** DJ-1 WT mouse cortical neurons were infected with the same constructs as above and after infection with lentivirus-expressing ATF4 shRNA (sh ATF4) or control shRNA (sh Ctrl) as control. Blots were probed with the indicated antibodies and quantified by Image J and graphed by Prism 7 (****p < 0.0001, two-way ANOVA with Tukey post-test, *n* = 3). **f** MTT assay are conducted as in **a** (*p < 0.05 M, two-way ANOVA with Tukey post-test, *n* = 5)

To determine whether or how expression of WT DJ-1 or its L166P mutant affect neuronal viability in the context of ER stress, DJ-1 WT (Fig. 8a) or KO (Fig. 8c) cortical neurons were infected with adenovirus-expressing WT or L166P DJ-1 and treated with Tun. ER stress-induced cell death was exacerbated by the expression of both WT and L166P DJ-1 (Fig. 8a, c). Interestingly, over-expression of L166P elicited ER stress-induced cell death that was similar to WT even though its expression was lower than DJ-1 WT (Fig. 8b, d). ER stress-induced neuronal death was accompanied by increase in cleaved capase-3 (Fig. 8b, d).

Finally, we tested whether ATF4 is involved in death induced by expression of DJ-1 WT and L166P under ER stress condition. ATF4 knockdown in cortical neurons significantly repressed both DJ-1 WT and L166P-induced cell death and cleaved caspase-3 level following ER stress (Fig. 8e, f). Notably, during ER stress, DJ-1 WT and L166P over-expression had no effect on P-PERK levels, indicating that high levels of DJ-1 WT or L166P do not induce ER stress leading to UPR activation (Fig. 8e). Together, our in vitro and in vivo data show that DJ-1 deficiency is protective in ER stress-induced cell death.

### DJ-1 deficiency ameliorates ER stress-mediated but not oxidative stress-mediated cell death

**Fig. 9:**
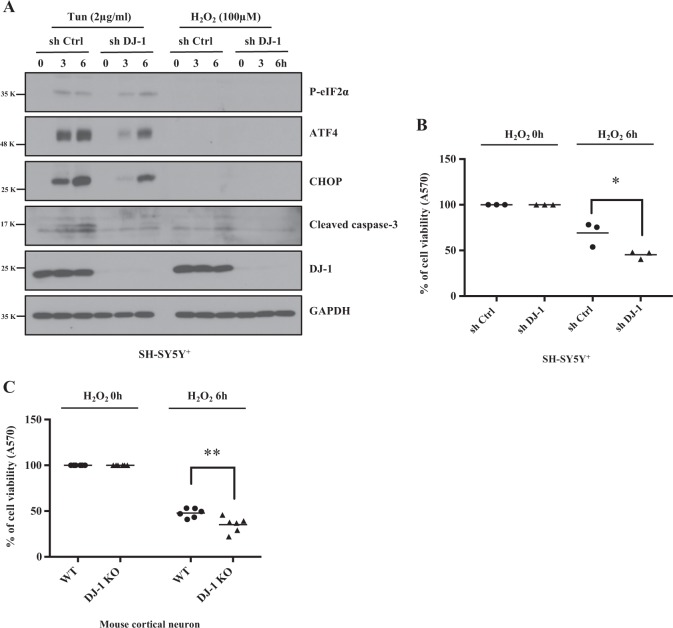
DJ-1 deficiency increases cell viability in ER stress, but not in oxidative stress. **a**, **b** Extracts of SH-SY5Y^+^ cells from sh Ctrl or sh DJ-1 after treatment with Tun for 3 to 6 h, or treatment with H_2_O_2_ for 3 or 6 h were used for immunoblotting with antibodies as indicated. GAPDH was used as a loading control. MTT assay was performed by using SH-SY5Y^+^ cells in sh Ctrl or sh DJ-1 (**b**), (*p < 0.05, two-way ANOVA with Tukey post-test, *n* = 3) or WT and DJ-1 KO mouse cortical neurons (**c**), (**p < 0.01, two-way ANOVA with Tukey post-test, *n* = 6) after treatment with H_2_O_2_ as indicated

DJ-1 has been reported to have antioxidant function and to protect cells from ROS-induced damage^31,32^. However, our results above show that DJ-1 deficiency has protective effects in ER stress. To help reconcile these opposing roles of DJ-1 in ER- and oxidative stress, we compared the effect of DJ-1 deficiency in both models. Control shRNA or DJ-1 KD SH-SY5Y^+^ cells were treated with either Tun or H_2_O_2_. As shown in Fig. 9a, whereas, P-eIF2α, ATF4, CHOP, and cleaved caspase-3 were induced under ER stress, induction of these proteins was not observed with oxidative stress (Fig. 9a). These results suggest that the cellular signaling triggered by ER- and oxidative stresses are distinct. DJ-1 KD SH-SY5Y^+^ cells and DJ-1 KO cortical neurons treated with H_2_O_2_ exhibited greater sensitivity to ROS-induced death when vs controls (Fig. 9b, c). These results are in contrast to data with Tun treatment that show increase resistance to ER stress-induced death (Fig. 6). Together, our results show that DJ-1 deficiency induces sensitivity to oxidative stress but protects against ER stress-induced cell death. Importantly, our data show that while both types of stress lead to cell death, the signaling pathway by which death is achieved is likely different.

## Discussion

DJ-1 regulates the level of ATF4 in response to ER stress. This evidence is summarized as follows: (1) DJ-1 loss reduces basal and ER stress-induced ATF4 induction. (2) Expression of WT or mutant DJ-1 leads to increased ATF4 levels. (3) ATF4 regulation is not due to DJ-1 transcriptional activity. Instead DJ-1 directly binds ATF4 transcripts increasing its stability. Surprisingly, DJ-1 loss is associated with neuronal survival under acute ER stress. This observation runs counter to most reports of DJ-1 acting as a pro-survival factor.

The potential role of ER stress in PD is growing. Hyper-activation of UPR is observed in PD patients and animal models^16^. However, the contribution of ER stress pathways to neurodegeneration is complex, with both pro-survival and pro-death pathways. For example, a dual role of ATF4 has been shown during ER stress. ATF4 translation is upregulated through increased P-eIF2α, in response to accumulation of misfolded proteins in the ER. This prevents mitochondrial damage by promoting the transcription of parkin^33^. However, induction of ATF4 can also lead to cell death through the induction of CHOP and caspase-3 activation^34^.

Our data demonstrate the complexity of ER response to neuronal death in that DJ-1 loss promotes neuronal survival in response to acute ER stress. DJ-1 appears to also modify ER stress pathways more directly through the regulation of ATF4. Our data suggest that DJ-1 normally stabilizes ATF4 transcripts via direct interaction and leads to increased ATF4 response.

The structural basis of interaction between DJ-1 and RNA is not clear because DJ-1 does not have classical RNA binding motifs. DJ-1 may bind GG/CC-rich sequences, allowing it to bind many RNA molecules^26^. Yet DJ-1 RNA targets are distinct from other RNA binding proteins such as FMR-1 and HuR^35,36^. DJ-1 likely binds to only a subset of RNA targets in a context dependent manner. A previous study reported that DJ-1 binds multiple RNA targets only upon oxidation^26^. In PD patients, DJ-1 binds with lower amount of mRNA of NADH dehydrogenase 2 (ND2) and NADH dehydrogenase 5 (ND5), leading to diminished expression of the protein, compared to healthy controls^9^. Modifying RNA turnover, rapid translation processes are important in controlling stress response and apoptosis^37^. We found that mutant L166P binds ATF4 mRNA similar to WT DJ-1. L166P is less stable and is rapidly degraded via ubiquitin/proteasome system^38,39^. However, in this study DJ-1 L166P did not act as a strict loss of function. DJ-1 L166P exists as a monomer in cells, which may allow it to gain ability to bind to ATF4 mRNA. For example, L166P has increased binding affinity to TTRAP when compared to WT, leading to cell death^40^. DJ-1 L166P also has increased binding affinity with mitochondrial Bcl-X_L_ than DJ-1 WT, leading to more Bcl-X_L_ expression in mitochondria than DJ-1 WT in response to UVB irradiation^41^. It is therefore possible that DJ-1 L166P may act as a gain of function by binding ATF4 mRNA to affect ER stress-induced apoptosis pathway, which may contribute to DJ-1 linked PD.

In acute ER stress, the stabilization of ATF4 is most associated with a downstream CHOP-caspase response leading to death. Fittingly, DJ-1 accelerates the ER stress response (as its absence limits) resulting in increased death. How is this model reconciled with the notion that DJ-1 loss leads to PD? The reason may lie in the complex nature of the ER response. In a more chronic setting, DJ-1 may stabilize an ER response that is more beneficial to DA health. Again, the context dependence of ATF4 in promoting survival or accelerating death is likely important. The type and/or strength of apical death initiators in model injury systems is also likely important. Models of oxidative stress are ATF4-CHOP-cleaved caspase-3 signaling independent. Indeed, we have shown other critical factors in DJ-1 mediated death including Pon2^42^. Finally, it is important to note that DJ-1 may also regulate a number of other targets/transcripts as mentioned above in addition to ATF4. How these potential interactions affect neuronal survival and whether it has any relation of ATF4 is unclear.

In the context of PD, neuronal death is likely determined by a number of factors including ER and oxidative stress. Previous studies have shown evidence of oxidative stress and damage in familial and sporadic PD brain tissues^43,44^. Even healthy individuals have increased oxidized proteins in the SN compared to other brain regions^45^. This indicates that susceptibility of SN to oxidative stress may contribute to selective neurodegeneration. Indeed, multiple familial PD genes including *PARK7* have been consistently implicated in the oxidative stress response or mitochondrial quality control pathways^46^. Environmental toxins implicated in PD act on mitochondrial complexes and create ROS^47^. While there is a bi-directional relationship between ER stress and oxidative stress, the exact mechanism(s) and their relationship to PD pathogenesis remain unclear. Tissue samples from PD patients show increased P-PERK, P-eIF2α, and ATF4 in the SNpc. Another study showed that MPP+ or 6-OHDA upregulates the expression of UPR proteins such as BIP, CHOP, ATF4, P-PERK, and P-eIF2α^16,48^. Here we show that DJ-1 null neurons are hypersensitive to ROS yet show a remarkable resistance to ER stress. In summary, DJ-1 may act as a key regulator between these two pathways that is context dependent. Therefore, ER stress and its crosstalk with the oxidative stress response may underlie the pathogenesis of PD. Experiments in chronic and aging models will be critical for future study.

## Materials and methods

### DJ-1 animals

DJ-1 KO mice were obtained from Dr. Tak W. Mak (The Campbell Family Institute for Breast Cancer Research, Ontario Cancer Institute, Toronto, Canada). The transgenic strains were maintained on a C57BL/6N background and genotyped as described previously^49–53^.

### Generation of DJ-1 adenoviruses

Adenovirus vectors-expressing human DJ-1 WT or L166P mutant were generated in pAdTRACK-CMV. Separate cytomegalovirus promoters drive the expression of GFP and DJ-1. Adenovirus was produced and tittered as described^54^.

### In vivo tunicamycin (Tun) administration

All animal experiments confirmed to the guidelines set forth by the Canadian Council for the Use and Care of Animals in Research (CCAC) and the Canadian Institutes for Health Research (CIHR) and with approval from the University of Ottawa Animal Care Committee. Tun (Sigma-Aldrich) was stereotaxically administrated above the SN in 1 years old mice brain using the following coordinates from bregma: −3.1 mm anterior, +1.1 mm lateral, and a depth of −4.4 mm. The mice received injection of Tun 0.4 ng/g. Tun was diluted in 0.9% saline at the concentration of 0.05 μg/μl. Mice in control group were injected with the same amount of 0.9% saline without Tun. Mice were sacrificed and perfused transcardially with 4% paraformaldehyde (PFA) one week post injection as previously described^11^. Assessment of dopamine neuron survival was performed blindly 1 week after the Tun injection by stereological assessment of immunohistochemical TH staining or cresyl violet staining counts as described previously^55,56^.

### Primary mouse cortical neurons and astrocyte culture

Primary mouse cortical neurons were obtained from 14.5 of DJ-1 WT and KO mice at embryonic days. Briefly, the cortex was dissociated by trypsin solution and mechanical triturating. After dissociation, the isolated cells were seeded at plates pre-coated with poly-d-lysine (PDL, 0.1 mg/ml) and maintained in complete neurobasal medium containing N2 and B27 supplements (Invitrogen) as described previously^57^. Drug treatments were initiated at 7 days after plating. Primary mixed glia cells were cultured from the cerebral cortices of 1–3 day old of DJ-1 WT and KO mice. The cortices were triturated into single cells by Pasteur pipettes in Minimal essential medium containing 10% heat-inactivated fetal bovine serum (FBS), 5% horse serum (HS), 0.11% gentamycin and 1% glutamax. The cells plated into 75-cm^2^ T-flasks (0.5 hemisphere/flask) pre-coated with PDL for 2 weeks. The microglia were removed from the flasks by shaking at 250 rpm for 30 min and were applied to a nylon mesh (40 μm) to remove other cells and cell clumps. Cells were plated and washed to remove unattached cells before being used in experiments. Following removal of the microglia, primary astrocytes were prepared using trypsinization.

### Midbrain culture on astrocyte

Mouse mesencephalic dopamine neurons on astrocyte culture was prepared following the published protocol^58^. Briefly, the primary cortical astrocyte was maintained in primary mixed glia cell culture medium as described above from 1 to 3 day old of DJ-1 WT and KO mice. Vigorous shaking is performed to remove other cells and microglia cells. At 90% confluence, astrocytes are trypsinized and plated on coverslip pre-coated with PDL. Upon reaching confluence on coverslip, the mesencephalic slice of DJ-1 WT or KO from 1 to 3days old mice brain were isolated, digested and dissociated. Then, the cells are plated at a density of 1 × 10^6^ cells on coverslip with astrocyte. The following day, a mitotic inhibitor is treated to inhibit astrocyte and microglia proliferation. After 7 days, cells were stained with TH antibody to count for TH-positive cells.

### Cell culture

SH-SY5Y cells were maintained in a mixture 1:1 of Ham’s F12 and Dulbecco Modified Eagle Medium (DMEM) supplemented with 10% heat-inactivated FBS, 1% antibiotic/anti-mycotic solution. Cell medium were replaced every 2 days and the cells were sub-cultured once they reached to 90% confluence. To differentiate SH-SY5Y cells, 30% confluent cells were treated with 10 μM all trans retinoic acid (Sigma-Aldrich) for 7 days. DJ-1 WT and KO MEFs were cultured in DMEM containing 10% FBS and 1% antibiotic/anti-mycotic solution.

### Generation of DJ-1 KD SH-SY5Y cells

SH-SY5Y cells were infected with 10 multiplicity of infection (MOI) DJ-1 shRNA or control shRNA lentiviral particles containing puromycin resistance gene following manufacturer’s recommendation (Santa Cruz Biotechnology). Infected cells were selected by culturing in 5 μg/ml puromycin (Sigma-Aldrich).

### Transfection and infection

In MEFs, transfections were performed using plasmids containing c-Flag pcDNA3, pcDNA3.1(+)-Flag-DJ-1 WT, pcDNA3.1(+)-Flag-DJ-1 L166P using Lipofectamine 2000 following manufacturer’s recommendation^59^. SH-SY-5Y^+^ cells and mouse cortical neuron were exposed to recombinant-adenovirus-expressing GFP only, GFP plus DJ-1 WT or GFP plus DJ-1 L166P mutant at 60 h after plating (MOI, 10–50) for 30 h. Mouse cortical neuron were infected with ATF4 shRNA or control shRNA lentivirus (1 MOI) for 7 days. Infected cells were used for western blots, RT-PCR, real-time PCR and 3-(4,5-dimethylthiazol-2-yl)-2,5-diphenyltetrazolium bromide (MTT).

### Antibodies and reagents

Antibodies against P-PERK, PERK, eIF2α, P-eIF2α (S51), ATF4, CHOP, cleaved caspas-3, caspase-3, BiP, and TrkB were purchased from Cell Signaling Technology. Mouse anti β-actin and flag were purchased from Sigma-Aldrich, Canada. DJ-1, GAPDH and GST antibodies were purchased from Santa Cruz. Secondary antibodies were from Thermo Scientific. Tun, Tg, and Actinomycin D were purchased from Sigma-Aldrich and were dissolved in DMSO at a stock concentration of 2 mg/ml, 1 mM, and 1 mg/ml, respectively. Hydrogen peroxide were purchased from Sigma-Aldrich at a stock concentration of 100 mM. All reagents above were diluted in cell culture media to the required concentration before addition to cells.

### Western blot

RIPA buffer (50 mM Tris–HCl, pH 7.4, 1% Triton X-100, 0.25% sodium deoxycholate, 150 mM NaCl, and 0.1% sodium dodecyl sulfate) containing protease inhibitors cocktail was used for for cells or mouse brain tissue lysis. The lysates were centrifuged at 13,000 rpm for 10 min at 4 °C, and the supernatant was collected. Proteins were resolved by SDS–PAGE, transferred to a nitrocellulose membrane (BIO-RAD) and immunoblotted with the indicated antibodies. Signals were then visualized by an enhanced chemi-luminescence (Thermofisher).

### Quantification of gene expression by RT-PCR and real-time RT-PCR

Cells were lysed using Trizol (ambion). RNA was insolubilized with 100% isopropanol and washed through 70% ethanol. cDNA was generated by using Avian Myeloblastosis Virus Reverse Transcriptase according to the manufacturer’s recommendation (Promega). ATF4, XBP-1 β-Acti,n or GAPDH relative mRNA levels were determined by RT-PCR or real-time RT-PCR. Real-time PCR reactions were performed with the Roter-Gene Q thermocycler (Qiagen). KAPA SYBR FAST qPCR master mix (Kapa Biosystems) was used to produce PCR products labeled with fluorescent during amplification reaction according to manufacturer’s instructions.

### RT- and real-time PCR primer sequences

#### Mouse ATF4

Forward: 5′-GAGCTTCCTGAACAGCGAAGTG-3′

Reverse: 5′-TGGCCACCTCCAGATAGTCATC-3′

#### Human ATF4

hATF4 Forward 5′-CTTCTTACAACCTCTTCCCC -3′

JW hATF4 Reverse 5′-GCTTCCTATCTCCTTCAGTG-3′

#### Mouse β-Actin

Forward: 5′-GGCTGTATTCCCCTCCATCG-3′

Reverse: 5′-CCAGTTGGTAACAATGCCATGT-3′

#### Human GAPDH

Forward: 5′-ATGGGGAAGGTGAAGGTCG-3′

Reverse: 5′-GGGGTCATTGATGGCAACAATA-3′

#### Mouse XBP-1

Forward: 5′-ACACGCTTGGGAATGGACAC-3′

Reverse: 5′-CCATGGGAAGATGTTCTGGG-3′

### Immunoprecipitation (IP)

IP was performed, as described^60^. Briefly, 1 mg of total cellular or brain tissue protein was extracted with IP buffer (25 mM Tri pH 7.6, 150 mM NaCl, 1% NP40, 1% Sodium deoxyclolate, 0.1% SDS). Goat anti-DJ-1 antibody N-20 or normal goat IgG as a negative control (Santa Cruz Biotechnology) was used for IP.

### RNA immunoprecipitation (RIP)

Cells were harvested in RIP buffer (150 mM KCl, 25 mM Tris pH 7.4, 5 mM EDTA, 0.5 mM DTT, 0.5% NP40, 100 U/ml RNAase inhibitor SUPERase, and 1× Protease inhibitor cocktail). The cell debris were pelleted by centrifugation at 13,000 rpm for 10 min. Antibody of goat anti-DJ-1 or goat IgG was added to supernatant and incubated for over night at 4 °C with rotation. Overall, 50 μl of protein G beads were added and incubated for over night at 4 °C with rotation. Beads were pelleted at 2000 rpm for 2 min, the supernatant was removed, and beads were resuspended in 500 μl RIP buffer and repeated for a total of three RIP washes. Beads were resuspended in 1 ml Trizol. ATF4 mRNA were determined by RT-PCR. DJ-1 protein isolated by the beads was detected by western blot analysis.

### In vitro RNA binding assays

Recombinant GST-tagged human DJ-1 protein (1 μg) or GST protein (1 μg) alone were mixed in RIP assay buffer described above with 10 μg of total RNA from mouse whole brain isolated by Trizol (Sigma) and incubated for overnight at 4 °C. The mixtures were incubated with 50 μl of glutathione sepharose beads for overnight at 4 °C. Beads were washed as described above. Beads were resuspended in 1 ml Trizol. ATF4 mRNA were determined by RT-PCR. GST proteins isolated by the beads was detected by western blot analysis.

### Cell viability assay

Mouse cortical neuron cultures were grown on 96-well plates at a density of 1 × 10^5^ cells/cm^2^. At 7 days of growth, cells were treated with 2 μg/ml Tun for 3, 12, 24, and/or 36 h or DMSO (0 h) as a vehicle control. MTT assay was performed following manufacturer’s recommendation (Roche). Absorbance at 570 nm was measured using a Biotek Hybrid Reader. The ratio of the absorbance of treated cells to that of the control cells was calculated and used to represent the percentage of cell survivals. SH-SY5Y^+^cells were grown on 96-well plates at a density of 2 × 10^5^ cells/cm^2^. SH-SY5Y^+^cells were treated with 2 μg/ml Tun for 3 or 6 h or DMSO (0 h) as a vehicle control, and 100 μM H_2_O_2_ for 3 or 6 h or H_2_O (0 h) as a vehicle control. MTT assay was performed described above.

### Immunocytochemistry

Cells were plated and grown on PDL-coated coverslips for analysis. Cells were fixed with 4% PFA overnight at 4 °C and permeabilized in 0.3% Triton X-100 PBS for 5 min. After blocking with 10% mouse serum in PBS for 1 h, cells were stained with the antibody indicated in the figure and mounted with Vecta shield including DAPI (Vector Laboratories). Final images were made using a confocal microscope (Zeiss AxioObsever D1)

### Flow cytometry

After treatment of Tun (2 μg/ml) for 24 h, MEFs or SH-SY5Y^+^ cells were labeled by APC conjugated annexin-V or PI, following the manufacturer’s protocol (eBioscience) and were fixed with 4% PFA for 1 h at 4 °C. The cells were analyzed using a FACS-BD LSRFFortessa (Flow cytometry core, University of Ottawa).

### Luciferase assay

For the luciferase assay, 1 μg of *firefly* luciferase vector pGL3 WT ATF4 promoter or pGL3 Basic (empty vector) as a negative control was transiently co-transfected together with 0.1 μg of *Renilla* luciferase vector into 1 × 10^4^ MEF DJ-1 WT or KO cells using Lipofectamine 2000 as indicated by the supplier (Invitrogen). At 48 h post-transfection, cells were treated for 6 h with 2 μg/ml Tun or DMSO (0 h) as a vehicle control. Firefly and Renilla luciferase were quantified by using the Dual Luciferase Reporter Assay as indicated by the supplier (Promega).

### Statistical analysis

Signals were quantified by Image J and data were analyzed and plotted using Prism 7, and expressed ± SEM (*n*), where *n* indicates the number of experimental repeats. The significance of difference between two groups was determined by paired two-tailed Student’s *t-*test. For comparison of multiple groups, the significance was determined by one-way ANOVA or two-way ANOVA followed by Tukey post-test using GraphPad Prism 7 (GraphPad Software, CA, USA).

## Supplementary information

Supplementary Data Figure Legend

Supplementary Data 1

Supplementary Data 2

Supplementary Data 3

Supplementary Data 4

Supplementary Data 5

Supplementary Data 6
