# Supplementary material for: DJ-1 modulates the unfolded protein response and cell death via upregulation of ATF4 following ER stress
Source: Cell Death Dis. 2019 Feb 12;10(2):135. doi: 10.1038/s41419-019-1354-2 (PMC6372623; doi:10.1038/s41419-019-1354-2)
Supplement: Supplementary file 1 — Supplementary Data Figure Legend [file 41419_2019_1354_MOESM1_ESM.pdf]

Supplementary data 1. DJ-1 modulates ER stress-induced ATF4 protein expression in MEFs A) DJ-1 WT and KO were collected after treatment with DMSO (0 h) as vehicle control or Tun for up to 12 h. Blots were probed with the indicated antibodies and quantified by Image J and graphed using Prism 7. Right panels of A) Data of A were obtained from the same conditions and averaged to compare P-eIF2α (****$p < 0.0001$, ***$p < 0.001$, **$p < 0.01$, *$p < 0.05$, two-way ANOVA with Tukey posttest, *n*=3), ATF4 (*$p < 0.05$, **$p < 0.01$, ***$p < 0.001$, two-way ANOVA with Tukey posttest, *n*=4) or CHOP (*$p < 0.05$, **$p < 0.01$, two-way ANOVA with Tukey posttest, *n*=3) protein levels between DJ-1 WT and KO for up to 12 h after normalization by β-Actin. B) DJ-1 WT and KO were collected after treatment with DMSO (0 h) as a vehicle control or Tg for up to 12 h. Blots were probed with the indicated antibodies.

Supplementary data 2. DJ-1 deficiency upregulates ER stress-induced IRE1 phosphorylation, XBP-1 mRNA splicing and cleaved ATF6 in MEFs, but not in mouse cortical neurons A) DJ-1 WT and KO were collected after treatment with DMSO (0 h) as vehicle control or Tun for up to 12 h. Blots were probed with the indicated antibodies and quantified by Image J and graphed using Prism 7. Right panel of A) Data were obtained from the same conditions and averaged to compare P-IRE1 protein levels between DJ-1 WT and KO for up to 12 h after normalization by β-Actin (*$p < 0.05$, **$p < 0.01$, ****$p < 0.0001$, two-way ANOVA with Tukey posttest, *n*=3). (B and C) Total RNA isolated from MEFs (B, *$p < 0.05$, two-way ANOVA with Tukey posttest, *n*=3) and mouse cortical neurons (C) respectively from DJ-1 WT and KO treated with Tun (2μg/ml) for up to12 h as indicated. Total RNA prepared was amplified by RT-PCR. PCR products were analyzed on 1.5% agarose gels and stained with ethidium bromide. DNA fragments derived from unspliced XBP-1 (uXBP-1) and spliced XBP-1 (sXBP-1) are indicated. D) DJ-1 WT and

KO were collected after treatment with DMSO (0 h) as vehicle control or Tun for up to 3 h in MEFs. Blots were probed with the indicated antibodies and quantified by Image J and graphed using Prism 7 after normalization by β-Actin (***p < 0.001, two-way ANOVA with Tukey posttest, *n*=3).

Supplementary data 3. Effects of over-expressed DJ-1 WT and pathogenic mutant L166P on basal or ER stress-induced ATF4 in MEFs A) DJ-1 WT and KO in MEFs were transfected for 40 h with pcDNA3-human DJ-1 WT, pcDNA3-human DJ-1 L166P or pcDNA3 (empty vector) only as control, then cells were collected. B) Experiments are conducted as in A, then treated with Tun as indicated concentration for 1 h or 3 h. Blots were probed with the indicated antibodies. C) DJ-1 WT mouse cortical neurons were infected with adenovirus expressing GFP plus Flag tagged DJ-1 WT, GFP plus Flag tagged DJ-1 L166P or GFP plus empty vector as a control. Blots were probed with DJ-1 or β-Actin antibodies and quantified by Image J and graphed using Prism 7 after normalization by native DJ-1 (*p < 0.05, **p < 0.01, ***p < 0.001, one-way ANOVA with Tukey posttest, *n*=3). D) DJ-1 WT mouse cortical neurons were infected with adenovirus expressing GFP plus Flag tagged DJ-1 WT, GFP plus Flag tagged DJ-1 L166P, GFP plus empty vector or non-infected (NI) as a negative control. Cells were fixed with 4% paraformaldehyde and assessed by direct microscopy and GFP positive cells were counted. (Scale bars, 200 μm)

Supplementary data 4. DJ-1 protein does not bind with ATF4 protein. Co-immunoprecipitation was performed in A) MEFs, B) DJ-1 WT mouse cortical neurons, C) SH-SY5Y^{+} cells and D) The SN of 3 months old mouse brain. DJ-1 protein was isolated by immunoprecipitation (IP) using DJ-1 antibody and blots were immunoblotted with indicated antibodies.

Supplementary data 5. DJ-1 deficiency inhibits ER stress-induced cell death. In MEFs, DJ-1 WT and KO were treated with Tun for 24 h. Cell death was analyzed by PI-positive staining (A) followed by flow cytometry. Induction of PI staining in 3 independent experiments by Tun for 24 h at indicated concentration was quantified with Kaluza and graphed using Prism 7 (*$p < 0.05$, two-way ANOVA with Tukey posttest, *n*=3). B) DJ-1 WT and KO cells in MEFs were collected after treatment with Tun (12 or 24 h) at indicated concentration. Blots were probed with the indicated antibodies. C) Tun or 0.9% saline (control) was stereotaxically administrated above the SN of 1 years old DJ-1 WT and KO mice brain. Sections of the SN at the level of the medial terminal nucleus was prepared from mice perfused 7 days post infection. Sections were stained with cresyl violet for counting in SN region. Representative images of cresyl violet positive cells in the SN were quantified using Stereo Investigator software. (*$p < 0.05$, two-way ANOVA with Tukey posttest, *n*=4 animals per group) (Scale bars, 100 μm)

Supplementary data 6. DJ-1 modulates ER stress-induced cleaved caspase-3 protein expression level in MEFs A) WT and DJ-1 KO MEFs were collected. Blots were probed with the indicated antibodies. B) Experiments were conducted as in A after treatment with DMSO (0 h) as vehicle control or Tun for up to 12 h. Blots were probed with the indicated antibodies. Right panel of B: Data was obtained from the same conditions and averaged to compare cleaved caspase-3 protein levels between DJ-1 WT and KO after normalization by β-Actin and quantified by Image J and graphed by Prism 7 (*$p < 0.05$, ***$p < 0.001$, ****$p < 0.0001$, two-way ANOVA with Tukey posttest, *n*=3). β-Actin was used as a loading control.
