# Supplementary figures and images for: DJ-1 modulates the unfolded protein response and cell death via upregulation of ATF4 following ER stress
Source: Cell Death Dis. 2019 Feb 12;10(2):135. doi: 10.1038/s41419-019-1354-2 (PMC6372623; doi:10.1038/s41419-019-1354-2)

# Supplementary data 1

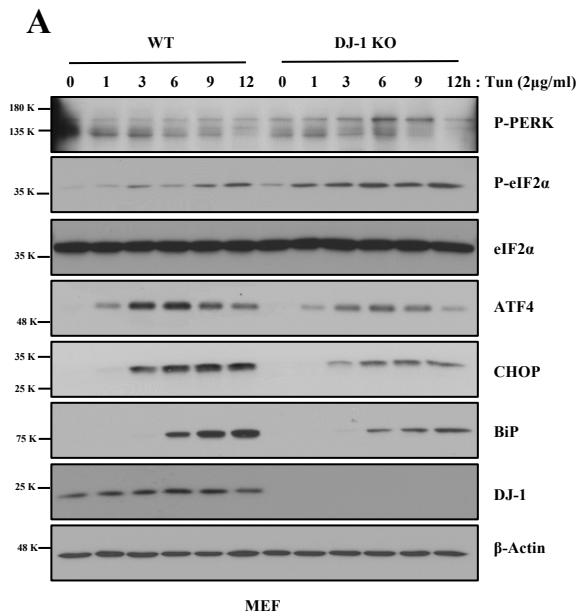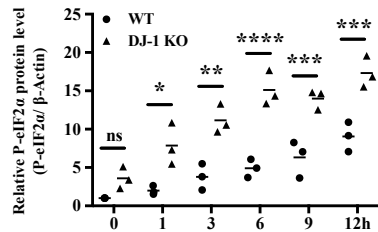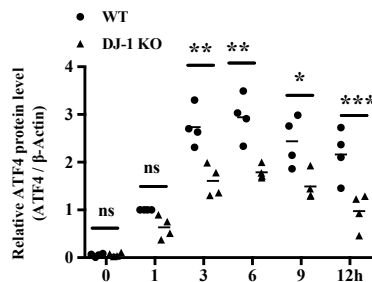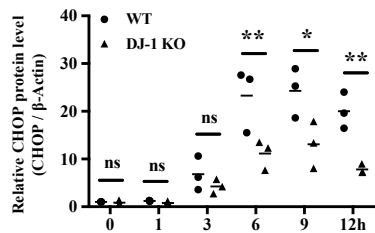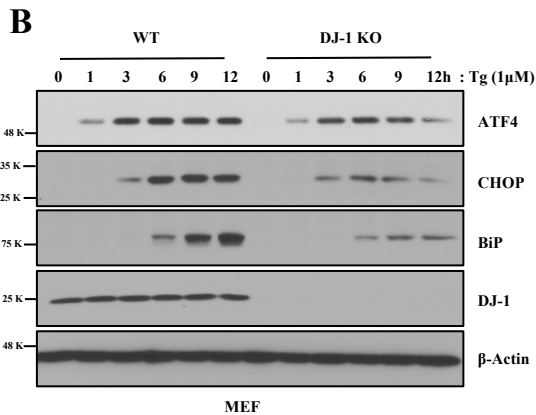

Supplement: Supplementary file 2 — Supplementary Data 1 [file 41419_2019_1354_MOESM2_ESM.pdf]

Supplementary data 2

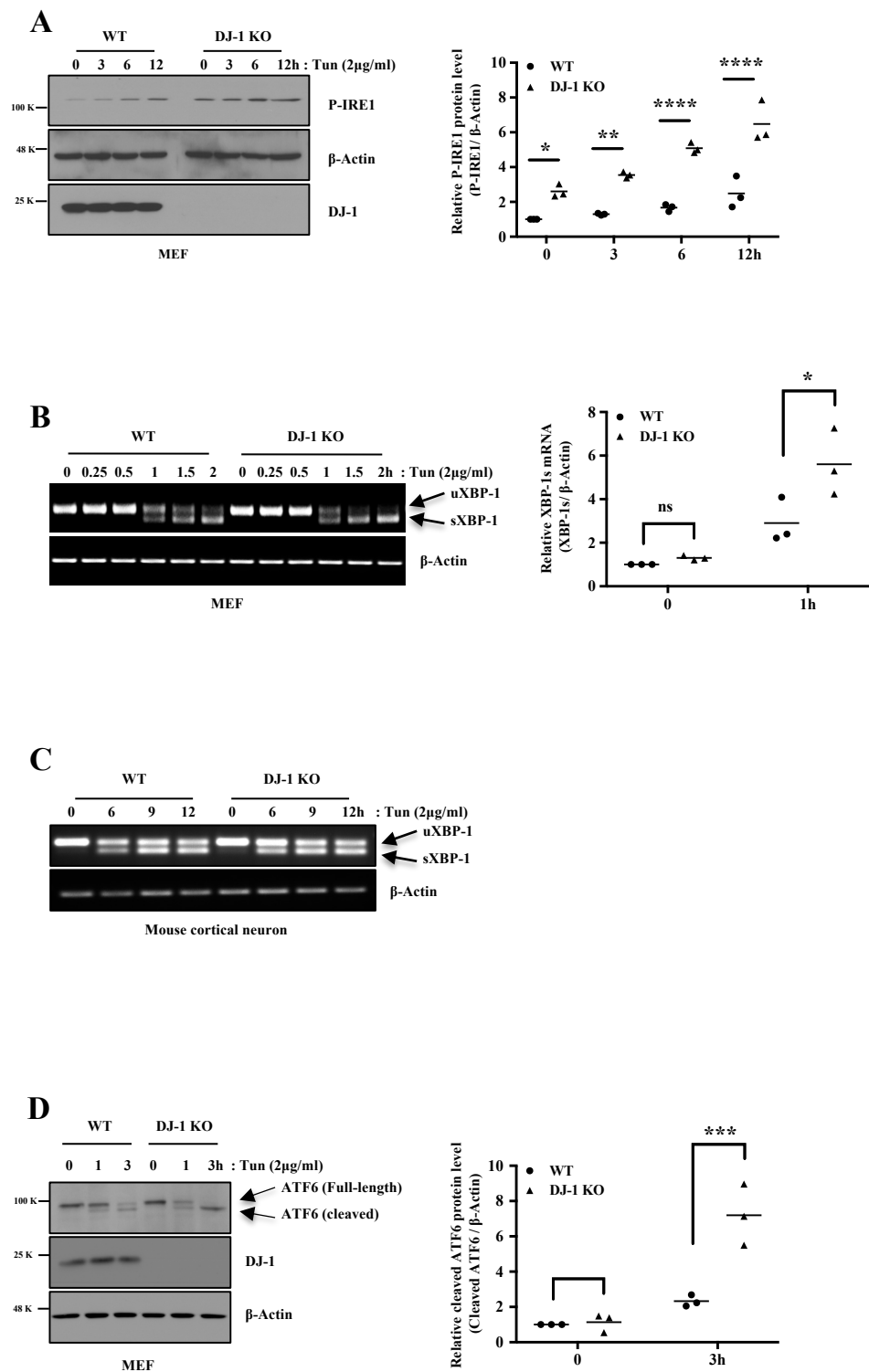

Supplement: Supplementary file 3 — Supplementary Data 2 [file 41419_2019_1354_MOESM3_ESM.pdf]

Supplementary data 3

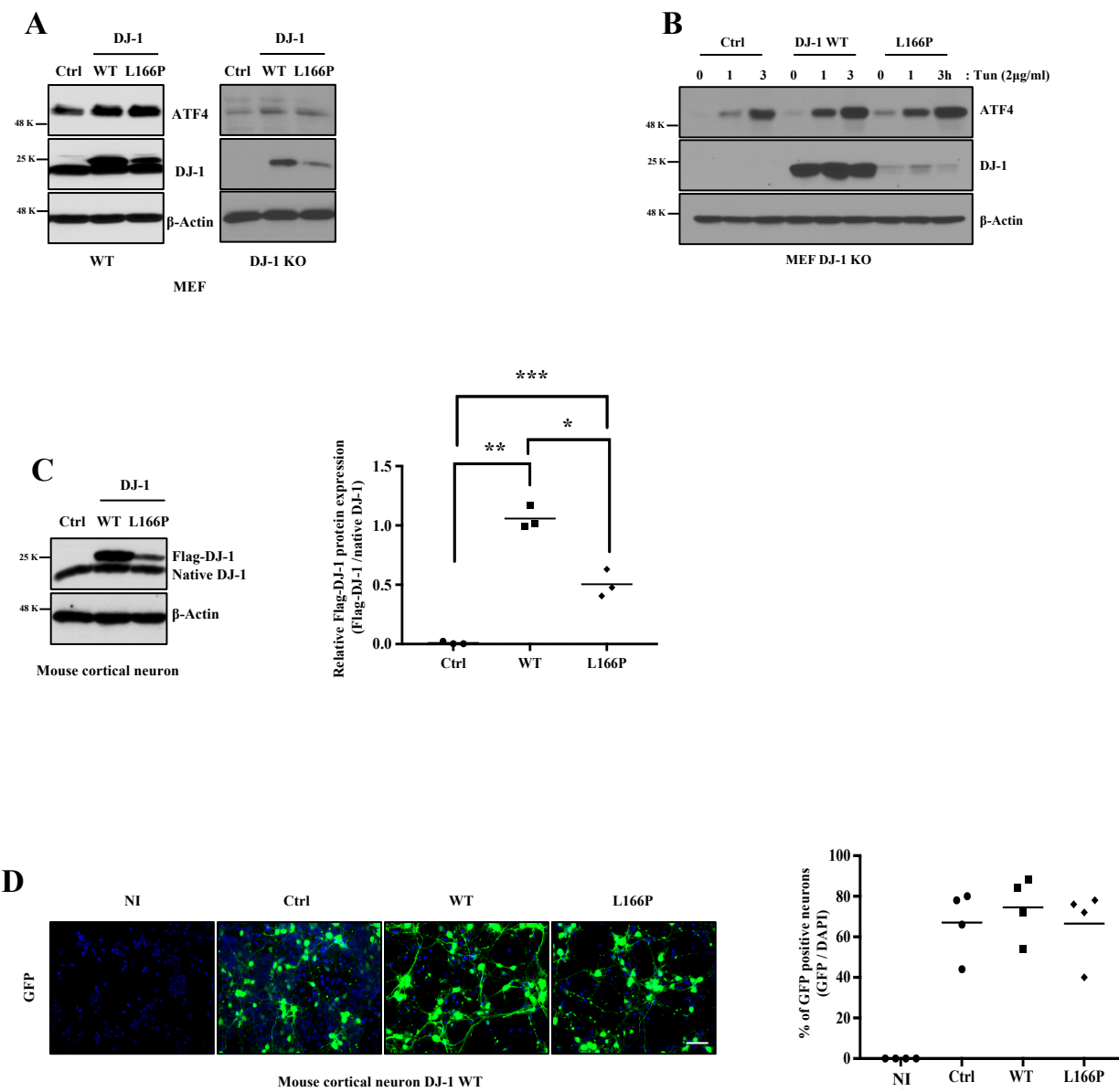

Supplement: Supplementary file 4 — Supplementary Data 3 [file 41419_2019_1354_MOESM4_ESM.pdf]

Supplementary data 4

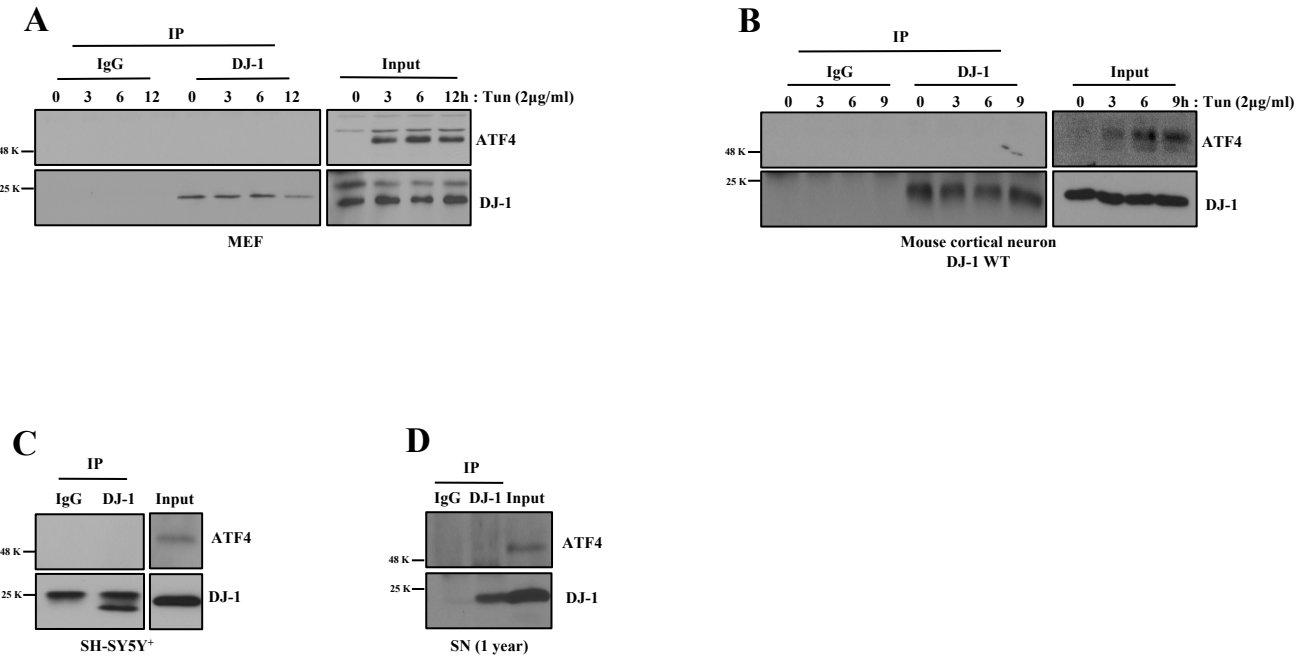

Supplement: Supplementary file 5 — Supplementary Data 4 [file 41419_2019_1354_MOESM5_ESM.pdf]

# Supplementary data 5

A

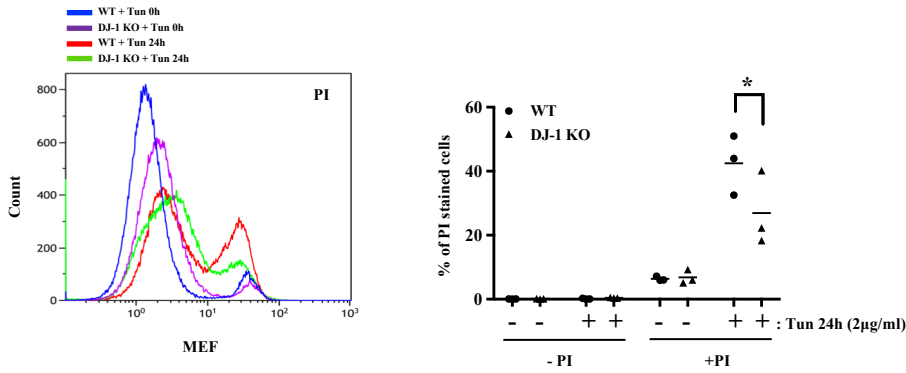

B

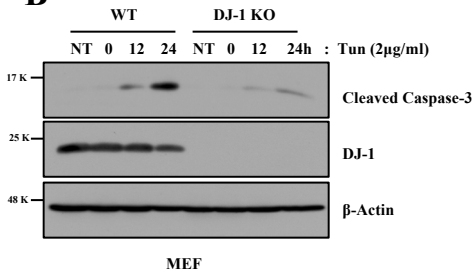

C

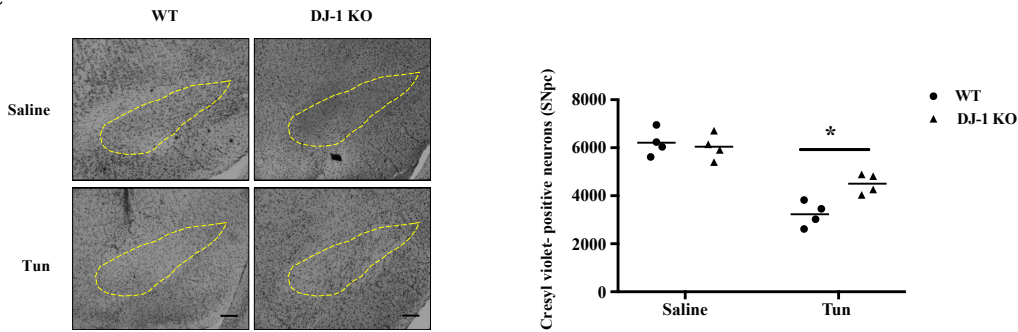

Supplement: Supplementary file 6 — Supplementary Data 5 [file 41419_2019_1354_MOESM6_ESM.pdf]

Supplementary data 6

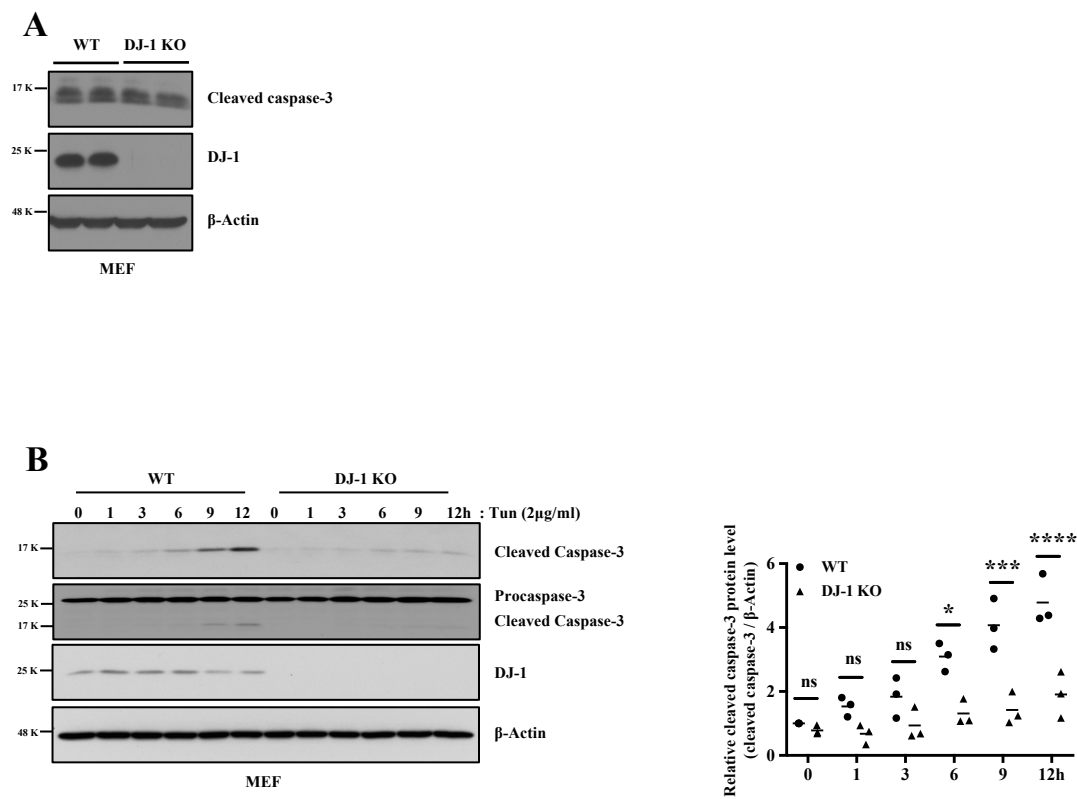

Supplement: Supplementary file 7 — Supplementary Data 6 [file 41419_2019_1354_MOESM7_ESM.pdf]
